# Integrated Human Surveillance Systems of West Nile Virus Infections in Italy: The 2012 Experience

**DOI:** 10.3390/ijerph10127180

**Published:** 2013-12-13

**Authors:** Christian Napoli, Antonino Bella, Silvia Declich, Giuliano Grazzini, Letizia Lombardini, Alessandro Nanni Costa, Loredana Nicoletti, Maria Grazia Pompa, Simonetta Pupella, Francesca Russo, Caterina Rizzo

**Affiliations:** 1 National Centre for Epidemiology, Surveillance and Health Promotion, National Institute of Health (Istituto Superiore di Sanità, ISS), viale Regina Elena, 299-00161 Rome, Italy; E-Mails: christian.napoli@iss.it (C.N.); antonino.bella@iss.it (A.B.); silvia.declich@iss.it (S.D.); 2 National Blood Centre, National Institute of Health, via Giano della Bella, 27-00161 Rome, Italy; E-Mails: giuliano.grazzini@iss.it (G.G.); simonetta.pupella@iss.it (S.P.); 3 National Transplantation Center, via Giano della Bella, 34-00161 Rome, Italy; E-Mails: letizia.lombardini@iss.it (L.L.); alessandro.nannicosta@iss.it (A.N.C.); 4 Department of Infectious, Parasitic and Immune-mediated Diseases, National Institute of Health (Istituto Superiore di Sanità, ISS), viale Regina Elena, 299-00161 Rome, Italy; E-Mail: loredana.nicoletti@iss.it; 5 Department of Prevention and Communication, Ministry of Health, via Ribotta, 5-00144 Rome, Italy; E-Mail: m.pompa@sanita.it; 6 Department of Public Health and Screening, Veneto region, Dorsoduro, 3493, 30125 Venice, Italy; E-Mail: francesca.russo@regione.veneto.it

**Keywords:** West Nile virus infections, epidemiology, surveillance, Italy

## Abstract

In Italy, a West Nile virus (WNV) surveillance plan was firstly implemented in 2008 and 2009 in two affected regions and, since 2010, according to a national plan, a WNV neuroinvasive disease (WNND) surveillance has to be carried out each year during the period 15 June–30 November, in those regions where WNV circulation has been demonstrated among humans, animals or vectors. Moreover, since WNV can be transmitted to humans even by blood transfusions and organ transplants obtained from infected donors, the national surveillance integrates the blood transfusions and organs transplant surveillances too. The paper describes the results of this integrated human surveillance in Italy in 2012. Overall, in 2012, 28 autochthonous confirmed cases of WNND were reported, 14 blood donations were found WNV positive by Nucleic Acid Amplification Test and no solid organ donors tested positive for WNV. Moreover, 17 cases of WNV fever were confirmed in Veneto region. When comparing the number of WNND cases reported to the surveillance system in previous 4 years (43 cases during the period 2008–2011), with those reported in 2012 an important increase was observed in 2012. The geographic distribution of human cases was consistent with the WNV circulation among animals and vectors. Moreover, the implementation of preventive measures for WNV transmission through blood components allowed the detection of blood donors positive for WNV, avoiding the further spread of the disease. Since surveillance strategies and preventive measures are based on the integration among human, animal and vector control activities, the Italian experience could be considered a good example of collaboration among different sectors of public health in a “one health” perspective.

## 1. Introduction

In Italy first human cases of West Nile virus (WNV) were detected in 2008 in two regions (Emilia Romagna and Veneto) [1,2]. Since then, a total of 43 human cases of West Nile Neuroinvasive Disease (WNND) have been reported in Italy until 2011 from five regions (Emilia Romagna, Friuli Venezia Giulia, Lombardia, Sardinia, Veneto) [3]. 

Following the first human cases detected in 2008, a WNV surveillance plan was implemented in the two affected regions and, after the increasing number of cases, in 2010 the Italian Ministry of Health (MoH) published the first national plan for WNND human surveillance at national level, that was updated in the following years [4,5,6]. According to the MoH national plan, the surveillance period was defined with the highest vector activity (15 July–15 November, later extended to 30 November). Moreover, since WNV can be transmitted to humans by vector and to recipients of blood transfusions and organ transplants obtained from donors, the national surveillance integrates the blood transfusions and organs and tissue transplant preventive measure. Since September 2008, after the first three human cases of WNND reported in the Emilia Romagna region, the National Blood Centre (NBC) adopted, in agreement with the regional health authorities, specific preventive measures in the areas where human cases were reported [7]. These preventive measures consisted in the introduction, during the vector activity period, of WNV Nucleic Acid Amplification Test (NAT) screening of all blood and blood components including peripheral, bone marrow and cord blood stem cell donations collected in the provinces reporting human cases of WNV infection and in a nationwide 28-day deferral for blood donors who had spent at least one night in these areas [8]. From 2009 to 2011, out of a total of 297,455 blood donations tested for WNV by NAT, 12 viremic donations were detected and discarded [7]. Regarding organs and tissues from cadaveric donors, since it was not possible to apply a suspension of the donation, the WNV NAT screening on all the donors resident or who have spent at least one night in the regions with a demonstrated human WNV circulation was introduced within 72 h from donation. From 2008 to 2011, out of a total of 496 organ donors tested for WNV, none of the donors was positive for WNV [9]. Nevertheless, in 2011, four cases of West Nile virus (WNV) transmission following a single multiorgan donation in north-eastern Italy were reported. The donor was tested for WNV by NAT prior to transplantation but it resulted negative [10]. In addition to the national plan for WNND, Veneto region, since 2010, implemented a regional surveillance system for West Nile fever (WNF) in humans [11]. Here we describe the results of the existing integrated WNV human surveillance in Italy (*i.e.*, WNND surveillance, WNF in Veneto region, blood donors and organ donors surveillance), in 2012.

## 2. Methods

### 2.1. West Nile Neuroinvasive Disease Surveillance

According to the national plan for human surveillance, “affected areas” are defined as provinces (NUTS-3) [12] where laboratory-confirmed WNV presence is demonstrated during the veterinary, entomological or human surveillance in previous year or during the current surveillance period (15 June–30 November). Identification of an affected area immediately triggers the definition of the “surveillance area” for the whole region (NUTS-2) [12] where the affected area is located (e.g., the WNV circulation in Venice province trigger the definition of surveillance area for the Veneto region). In the “surveillance area” passive human surveillance has to be set up, requesting physicians to report all possible, probable and confirmed WNND cases using a modified European case definition [13]:

1. Possible: Patients with fever ≥38.5 °C and neurological symptoms (encephalitis, meningitis or Guillain-Barré syndrome or acute flaccid paralysis).
2. Probable: A possible case + anti-WN IgM positive in blood and/or anti-WN IgG positive in blood.
3. Confirmed: Possible/probable case and at least one of the following laboratory criteria: Viral isolation in blood or cerebrospinal fluid (CSF); anti-WN IgM positive in CSF; Polimerase Chain Reaction (PCR) positive in blood, CSF or urine; seroconversion (4 fold increase in anti-WN antibodies between different samples collected during the acute and convalescent stage of the disease); confirmed presence of anti-WN antibodies in blood by neutralization test.

Compared to EU case definition, the national case definition included only neurological symptoms, since the fever by itself would have been a too-wide-criteria, and defined also “possible case”, to help clinicians to identify patients that should be tested for WNV. Moreover, we considered for the case confirmation two additional tests: a PCR positive in urine, since this method has been demonstrated to be a useful diagnostic tool that enables the detection of WNV genomes, even after prolonged times post-infection [14,15,16,17]; and the seroconversion test, since the detection of WNV-specific neutralizing antibody activity has been demonstrated coincident with detection of seroconversion by commercially available IgM and IgG [18].

All possible cases have to be notified by the regional authorities to the MoH and to the National Centre for Epidemiology, Surveillance and Health Promotion-National Institute of Health (CNESPS-ISS) using a specific form to be filled in an internet-based platform. 

### 2.2. West Nile Virus Surveillance in Blood Donors

In 2012 the National Blood Center, on the basis of the estimated residual risk of transfusion transmission of WNV—calculated on the 2011 data according to the mathematical model of Biggerstaff and Petersen [19]—decided to introduce, as a preventive measure, from 15 July to 30 November, WNV NAT testing of all blood and blood component donations collected in the provinces with demonstrated human circulation of WNV in the previous or current year. NAT testing could be performed by mini-pool (MP) (size 6) technique, until the notification of a new human case of WNND or a detection of a NAT positive donor in the current year; after this NAT testing should be continued by single test technique (ID). The preventive measures included also nationwide the 28-day deferral of blood donors who had been at least one night in the affected areas [8]. 

### 2.3. West Nile Virus Surveillance in Solid Organs Donors

Since 2008, the Italian National Transplant Network (INTN), in collaboration with the regional health authorities, started an epidemiological surveillance programme in order to detect WNV in organ donors in Italy [10]. In addition to this epidemiological monitoring, the INTN decided to perform NAT within 72 h of donation on all donors living in regions reporting human cases of WNV infections. These measures were carried out from 15 July to 30 November 2012 in order to prevent WNV transmission from organ and/or tissue donations to recipient patients [20]. WNV NAT test was carried out on the blood sample collected from donor and performed by single test technique (ID).

### 2.4. West Nile Fever Surveillance System in Veneto Region

Since 2010, the Veneto region has implemented the activities of the National Surveillance Plan and intensified the surveillance of human cases of autochthonous WNV infection by activating an enhanced regional surveillance plan for WNF. According to the regional surveillance plan, cases of WNF were defined as being over 15 years-old, having fever ≥38.5 °C (or history of fever in the last 24 h) for a period no longer than seven days occurring from 15 July to 30 November, no history of recent travel to tropical countries, and absence of other concomitant diseases which could account for the febrile illness [11,21]. All confirmed cases have been notified by the regional authorities to the MoH and to the CNESPS-ISS using the specific internet-based platform built up for the WNND national surveillance.

### 2.5. Surveillance System Integration

A specific password-protected internet-based platform permits the local or regional health authorities to fill an electronic questionnaire for each possible cases of WNND and confirmed cases of WNF and to update the entry as more information becomes available. The regional reference laboratory (or the hospital laboratory) has to confirm the case according to the laboratory criteria reported in the case definition. If no confirming tests are available at the regional level, patient’s specimens should be sent to the National Reference Laboratory at ISS for confirm.

The CNESPS-ISS is responsible for the epidemiological surveillance and analysis of data provided. The password-protected access to the database is allowed to the NBC and to the INTN, that—on the basis of the uploaded data concerning the human circulation of WNV—implement precautionary measures on blood donation and transplant activities.

Moreover, human surveillance is integrated with veterinary and entomological surveillance too; in fact, as stated above, regions where human surveillance must be carried out are those where WNV-positivity (in human, animals or vectors) have been described in previous year or during the current surveillance period. In Italy, veterinary and entomological surveillances of WNV include surveillance on resident birds species, migratory bird populations, horses, and vectors and it is coordinate by the National Reference Centre for the Study of Foreign Diseases (CESME) [22].

## 3. Results

### 3.1. West Nile Neuroinvasive Disease Surveillance

**Figure 1:**
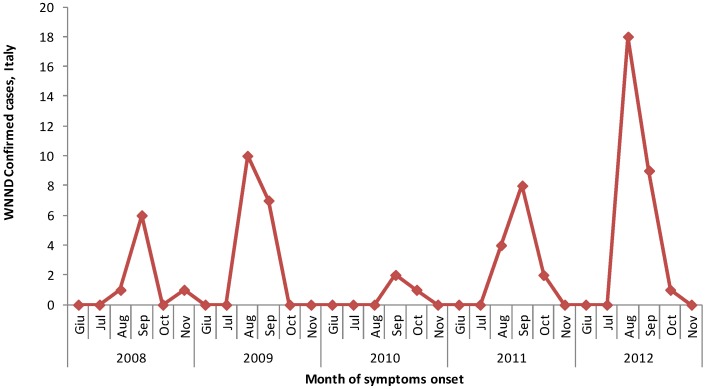
WNND confirmed cases by date of symptoms onset, Italy 2008–2012.

From 15 June to 30 November 2012, 99 possible cases of WNND were reported, of which 28 were confirmed. Table 1 shows the distribution of WNND confirmed cases by age and region/province of exposure. All 28 cases did not report travelled history during the incubation period. The median age was 69 years (range: 10–90 years). Overall, 61% were male. August was the peak month in 2012 (Figure 1). 

**Table 1:** WNND confirmed cases by region/province of exposure and age group, Italy 2012.

Region/Province	Age Groups					Total
	≤14	15–44	45–64	65–74	≥75	
Basilicata						
*Matera*			1			1
Friuli V.G.						
*Gorizia*					1	1
*Pordenone*		1	1			2
*Udine*			1			1
Sardegna						
*Oristano*					2	2
Veneto						
*Treviso*		1	1	2	2	6
*Venice*	1	1	3	4	6	15
Total	1	3	7	6	11	28

**Figure 2:**
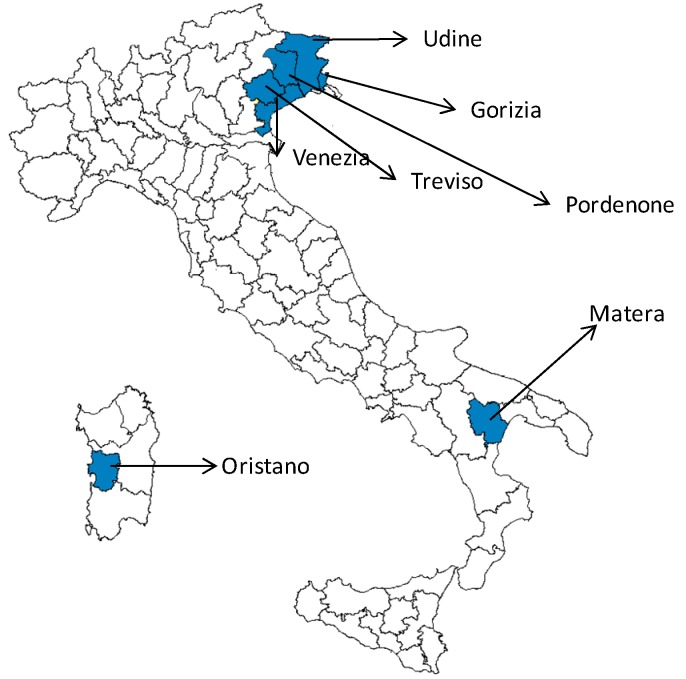
Provinces with confirmed human cases of WNND, Italy 2012 (7 provinces, 28 cases).

Figure 1 shows the distribution of WNND human cases by month of symptom onset, during the period 2008–2012 [3]. The majority of WNND cases in 2012 were reported from the provinces of Venice (15 cases) and Treviso (6 cases), both located in Vento region; Friuli Venezia Giulia region reported 4 cases, Sardinia 2 cases, and a new region in Southern Italy (Basilicata) reported one case in Matera province (Figure 2). 

The majority of cases reported symptoms of encephalitis (68%, 19/28), followed by meningitis (14%, 4/28), polyradiculoneuritis (11%, 3/28), and other neurological symptoms (7%, 2/28).

None of the patients had history of vaccination against other arboviruses, but one, that reported to be vaccinated against tick-borne encephalitis and Japanese encephalitis. This data is clinically relevant because the main weakness that limits the serological methods widely used in routine laboratory (*i.e.*, enzyme-linked immunosorbent assays, ELISAs and immunofluorescence, IF) is the broad antigenic cross-reactivity that exists between all flaviviruses [16,23]; for this reason, any single serological sample tested positive must be confirmed by the more specific tests, *i.e.*, neutralization test. In particular, the patient had a WNV encephalitis confirmed by serological neutralization test; moreover, IgM and IgG tested positive in CSF.

**Figure 3:**
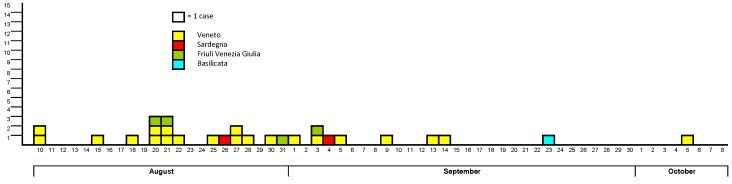
WNND confirmed cases by date of symptoms onset, Italy 2012.

The onset of cases is included in the period of 10 August–5 October (Figure 3).

All WNND cases were hospitalised; only one case—male 82 years old, with encephalitis and not known underlying diseases-died, corresponding to a case fatality rate of 3.6%.

### 3.2. West Nile Virus Screening in Blood Donors

WNV NAT screening at the beginning of 2012 surveillance period was performed in the following provinces where the human circulation of WNV was demonstrated in the previous year: Udine (Friuli Venezia Giulia region), Treviso, Belluno and Venice (Veneto region) as well as all the provinces of the Sardinia region. Later, between August and September, after four human cases of WNND reported in the provinces of Udine, Gorizia and Pordenone (Friuli Venezia Giulia region), the same preventive measures were extended to all provinces of the Region and, at the end of September, in the province of Matera (Basilicata region) when one WNND case was reported.

In 2012, from 15 July to 30 November, overall 116,255 blood donations underwent to the WNV screening by NAT testing. About half of them has been tested by MP NAT and the other half by ID NAT. Overall, 14 donations were found WNV NAT positive: 11 in the Venice province and 3 in the Treviso province. The 14 WNV NAT positive donors were asymptomatic at pre-donation questionnaire and physical examination. None of them reported flu-like symptoms in the post-donation interview. The incidence was 1.2 positive donations per 10,000. The residual risk of WNV transmission through blood transfusion was estimated at 0.26/10,000 donations. 

### 3.3. West Nile Virus Screening in Solid Organs Donors

From June 15 to November 30, all organs donors coming from those regions reporting human cases of WNV infections were tested for WNV NAT within 72 h from donation. A total of 162 organ donors were tested by WNV NAT in Veneto, Friuli Venezia Giulia and Sardinia region. From August 2012, after that a human case of WNND was reported in the provinces of Matera, the WNV NAT was performed also in Basilicata region. None of the donors tested were positive for WNV. 

### 3.4. West Nile Fever Surveillance System in Veneto region

From 15 June to 30 November, 17 confirmed cases of WNF were reported to the MoH and CNSPS-ISS by the Veneto regional authorities. The median age was 62 years (range: 45–79 years) and 76.5% were male. None of the cases had history of travel to affected areas 3 weeks before the symptoms onset, but one travelled to Bosnia. The cases were reported from the provinces of: Venice (13 cases), Vicenza (2 cases), Treviso (1 cases) and Padua (1 case). The onset of cases is included in the period 17 July–28 September; no deaths were reported.

## 4. Discussion

In Italy, the incidence of the WNND increased two-fold from 2008 to 2009; this trend probably reflects the great attention played, at the beginning of the surveillance, to the new introduction of a non-endemic disease in Italy. As a matter of facts, although, at that time, the human WNV surveillance plan had been implemented only in two regions (Veneto and Emilia Romagna), in 2009 a new region (Lombardia) occasionally reported two human cases, with the distribution of cases apparently moving from east to west [3]. In 2010, after the publication of the national plan for WNND human surveillance the incidence rate decreased four-fold, since in that period the circulation of the virus was lower and more delayed in the two affected regions [24]. Both this lower WNV circulation and the prevention measures implemented resulted in the absence of new cases for Emilia Romagna in the following years and a reduction of cases in Veneto in 2010. But, a new pick in Veneto and additional cases in new regions (Friuli Venezia Giulia and Sardinia) lead to a new increasing incidence in 2011, with a starting southward expansion of cases distribution. 

In 2012 the maximum number of cases per year was reached in Italy, with cases occurring both in the same provinces affected during the previous year (Udine, Oristano, Treviso, Venice) and in new ones (Gorizia, Matera and Pordenone). The southward expansion of cases distribution was confirmed also this year with the identification, for the first time, of a WNND case in a Southern region (Basilicata). Although Basilicata reported a human cases for the first time in 2012, some outbreaks of WNV infections in horses in Matera province (Basilicata) were already reported by CESME in 2011 [25]; therefore, since the beginning of 2012, Basilicata was included among the surveillance areas. After this case of WNND, all prevention measures were activated in Basilicata: vector control, prevention of mosquito bites, screening for exposed workers, control procedures related to blood and solid organ donation. No other cases, neither possible nor probable, were reported from Basilicata.

In 2012, August was the peak month as in 2009, while in 2008, 2010 and 2011 the peak was in September. 

From a clinical point of view, more than 80% of the total of WNV infected individuals is asymptomatic [26,27]. Among symptomatic infections, the vast majority of symptoms ranges from a mild flu-like syndrome, WNF, to severe WNND. WNND (mainly encephalitis, but also meningitis, meningoencephalitis, or acute flaccid paralysis) involves less than 1% of the infected patients [23,27]. In Italy in 2012, the large part of WNND was reported as encephalitis, but no acute flaccid paralysis were registered. 

If we consider the period 2008-2012, in Italy the total case fatality rate (CFR) of about 10% (8/71, 11.3%) is included in the range of those reported from other countries: 8% in Israel in 2005–2010 [28]; 9% in US in 2012 [29]; 15% in Greece in 2010–2011 [30]. Nevertheless, the annual CFR varied largely during the surveillance period in Italy: from 0% in 2008 and 2010 up to 3.6% in 2012, 16.7% in 2009 and 28.6% in 2011 [3]. Whether the high CFR was due to a high virulence of the circulating strains, increased host susceptibility or under-detection of milder cases remains undefined [31]. From an epidemiological point of view, undeniably, higher CFR may be related to an overestimation due to the more likely reporting of the most severe cases. On the contrary, lower CFR may be related to the younger age of the patients, as in 2010, when the median age was 46.5 years [3]; as a matter of facts, although risk factors for developing severe clinical illness are largely unknown, it has been demonstrated that an exaggerated innate immune responses lead to the more severe cases, and the role of aging in enhancing the WNV-induced innate immune response has been recently clarified [32,33]. What is particularly interesting is that, in 2012, while the number of cases reached the highest pick, the CFR was low. Anyway, lower CFR were also reported in other experience: during the first major WNF epidemic in Europe, experienced in Romania in 1996, when 393 patients with WNF infection were identified, of whom 352 had WNND with a CFR of 4.3% [34]. 

Only WNV lineage 1 human infections have been described in Italy until 2011, when both lineage 1 and 2 were detected in human cases too [3,31]. Since then, co-circulation of lineage 1 and 2 has continued to be confirmed in both humans and animals in Italy [22,35].

The measures for the prevention of WNV transmission through blood components implemented in Italy between 2008 and 2012 appear to have effectively improved the safety of the blood supply as no cases of WNV transfusion transmission have been reported so far by the National Haemovigilance System. In particular, in 2012, the screening by NAT testing allowed the identification of 14 donations WNV NAT positive out of 116,255 blood donations tested; with a rate of detection of WNV-positive donation being 1 out of 8,304 donations. Moreover, considering that most of these positive donations are systematically fractionated into three different blood components (red cells, buffy coat/platelets and plasma), a considerable number of blood recipients, often immunocompromised, were spared exposure to these potentially infectious units [7]. 

Therefore, the strategy of implementing WNV NAT screening of blood donations only in specific geographical areas, where WNV circulation was demonstrated, is considered the right balance, in term of cost-effectiveness, between financial resource available and protection of public health [7].

Measures undertaken in transplantation field aimed at recognizing the possible early positivity of the donor in order to intervene, for example through the administration of immunoglobulins, on the control of the neuroinvasive diseases in immunocompromised patients. Obviously, these measures are planned to attend patients rather than to prevent the transmission of WNV infection. 

It should be noted that, in Italy, all human surveillance activities and the related prevention measure activated in blood donation and organ transplantation are based on an integration with veterinary and entomological surveillances too. In fact, results of these surveillances contribute in the identification of affected areas, having a great impact on human surveillance system.

The animal and vector surveillances are coordinated by CESME. In 2012, outbreaks in horses for a total of 63 cases of which 15 with neurological symptoms (from Veneto, Sardinia, Friuli Venezia Giulia, Lazio regions) were confirmed; 16 alive plus five dead wild birds were PCR positive for WND symptoms (from Veneto, Sardinia, Friuli Venezia Giulia regions); five outbreaks in poultry farms (as an alternative to surveillance of synanthropic species) were confirmed in Basilicata region; finally, 14 mosquito pools (*Culex pipiens*) were positive by RT-PCR (from Veneto, Friuli Venezia Giulia and Sardinia regions) [22]. 

In 2013, integrated human, entomological and animal surveillance continued in Italy in order to monitor the spread of WNV and to implement control measures for blood transfusions and organ donations and to control and prevent transmission of the disease in humans.

## 5. Conclusions

Identification of human cases of WNND in Italy started in 2008, when a human surveillance system was implemented in affected areas where the WNV circulation was demonstrated among animals and vectors. Since then, cases of WNND have been reported every year in Italy, with a pick of incidence in 2012. The geographic distribution of human cases resulted consistent with the WNV circulation among animals and vectors: in 2012 all regions where WNV circulation in animal and vectors has been demonstrated, but one (e.g., Lazio region), reported human cases. 

These findings confirm the crucial role of an integrated human, animal and vector surveillance in order to timely set up preventive measures, such as the early detection of infected blood donors. In 2012, 14 asymptomatic blood donors coming from affected areas, tested positive for WNV; this evidence introduced the preventive measures for blood donors avoiding the further spread of the disease among blood recipients.

In conclusion, the Italian experience represents a good example of collaboration among different sectors of public health (human, veterinary, entomologists and blood and organ donation authorities) in a “one health” perspective [36]. It could serve as an effective rapid risk assessment for other vector-borne diseases too (e.g., chikungunya and dengue fever) [37]. 
